# Recurrent Urinary Stone Formers: Imaging Assessment and Endoscopic Treatment Strategies: A Systematic Search and Review

**DOI:** 10.3390/jcm13123461

**Published:** 2024-06-13

**Authors:** Charalampos Mavridis, Athanasios Bouchalakis, Vasiliki Tsagkaraki, Bhaskar Kumar Somani, Charalampos Mamoulakis, Theodoros Tokas

**Affiliations:** 1 Department of Urology, School of Medicine, University General Hospital of Heraklion, 71110 Heraklion, Greece; ch.mavridis@uoc.gr (C.M.); mpouxalakisth@gmail.com (A.B.); mamoulak@uoc.gr (C.M.); 2 Campus of Voutes, University of Crete Library, 70013 Heraklion, Greece; tsagkarv@uoc.gr; 3 Department of Urology, University Hospitals Southampton, NHS Trust, Southampton SO16 6YD, UK; bhaskarsomani@yahoo.com; 4 Training and Research in Urological Surgery and Technology (T.R.U.S.T.)-Group, 6060 Hall in Tirol, Austria

**Keywords:** stone formers, urolithiasis, nephrolithiasis, stone recurrence, kidney stones, PCNL, URS, RIRS

## Abstract

**Background/Objectives**: Nephrolithiasis is a heterogeneous disease with a high prevalence and recurrence rate. Although there has been much progress regarding the surgical treatment of stones, a standardized follow-up, especially in recurrent stone formers (SFs), has yet to be decided. This fact leads to the overuse of computed tomography (CT) scans and many reoperations in patients, thus increasing their morbidity and the financial burden on the health systems. This review systematically searched the literature for original articles regarding imaging strategies and endoscopic treatment for patients with recurrent urolithiasis, aiming to identify optimal strategies to deal with these patients. **Methods**: We systematically searched the Medline database (accessed on 1 April 2024) for articles regarding imaging modalities and endoscopic treatment for patients with recurrent urinary tract lithiasis. **Results**: No specific follow-up or endoscopic treatment strategy exists for patients with recurrent urolithiasis. CT scan was the imaging modality most used in the studies, followed by X-ray, ultrasonography, and digital tomosynthesis. A transparent algorithm could not be identified. Percutaneous nephrolithotomy (PCNL), retrograde intrarenal surgery (RIRS), and ureteroscopy (URS) were used in the studies for endoscopic treatment. PCNL showed the best stone-free (SFr) rate and lowest hazard ratio (HR) for reoperation. RIRS showed superiority over extracorporeal shockwave lithotripsy for recurrent SFs, but fragments over 4 mm increased the recurrent rate. URS has an increased HR for reoperation for bilateral stones. **Conclusions**: The heterogeneity of urolithiasis leaves urologists without a standardized plan for recurrent SFs. Thus, each patient’s follow-up should be planned individually and holistically. Pre-stenting is not to be avoided, especially in high-risk patients, and SFr status needs to be the aim. Finally, CT scans should not be generally overused but should be part of a patient’s treatment plan. Prospective studies are required to define SFr status, the size of significant residual fragments, and the modalities of intervention and follow-up.

## 1. Introduction

Urolithiasis is a vital symptom—a complication—and is often considered a disease. The prevalence and incidence of lithiasis seem to increase significantly over time, especially in countries where data are correctly recorded. The prevalence of urolithiasis has been documented to have increased in Spain from 0.1 to 10, while 9.8% of adults above 45 years report the disease in France [1]. In Greece, specifically Thebes, one of the highest one-year prevalence rates of 15.2% has been recorded. Regarding the incidence, the example of Germany is typical, where it has increased 6-fold in almost 20 years [1]. The gap between men and women seems to be closing nowadays, while the incidence with age increases [1,2]. Recurrent stone formation is another significant concern affecting patients and healthcare providers. It is a complex issue that requires a multidisciplinary approach. The condition is characterized by the recurrence of stone formation in the same or different parts of the urinary tract despite the successful treatment of previous stones.

The pathophysiology of urinary tract lithiasis is a complex challenge for the scientific community. The traditional theory of lithogenesis, which is based on the supersaturation and subsequent nucleation of crystals and growth aggregation, does not seem to be verified in many cases [3]. Current data suggest the disease is multifactorial and systemic, involving genetic and environmental factors. Specific genetic variants could increase the risk of stone formation. Environmental factors, such as dietary habits and hydration levels, also play a role in stone development [3]. The presence of other medical conditions, such as diabetes and obesity, can further complicate the risk of stone formation. This multifaceted nature of the disease underscores the importance of a multidisciplinary approach to its management.

Several factors have been proposed to increase the likelihood of recurrent stone formation. Specific genetic variants are associated with an increased risk, and recent examples include the upregulation of the vitamin D receptor and the downregulation of claudin 14 [4]. These alterations lead to hypercalciuria, which favors lithogenesis [4]. Lately, there has been considerable discussion about Nutrigenomics. For example, acetic acid (vinegar) appears to reduce urinary calcium and increase urinary citrate, probably by promoting the expression of some specific microRNAs [4]. A diet rich in oxalates, calcium, and phosphorus, inadequate hydration, and medical conditions such as diabetes, obesity, hypertension, cardiovascular and chronic kidney disease, and gout can contribute to stone formation [1,2,3]. However, targeted randomized studies still need to be conducted to improve fluid intake and diet recommendations [2]. A recent meta-analysis showed that the main risk factors for recurrent lithiasis are uric acid stones, stones in the pelvis or lower pole, asymptomatic non-obstructive stones, previous renal colic before the first confirmed case, previous interventions for a stone, white race, and hypertension [1].

One of the main problems in managing patients with stone relapses is their cumulative radiation exposure and general anesthesia sessions. The role of healthcare providers in managing this aspect is crucial, as there are no clear guideline recommendations. One in five patients with a single episode of urolithiasis receives a high dose of radiation within the first year of follow-up [5]. Data show that 10% of patients with urolithiasis receive radiation above safety due to their submission to kidney–ureter–bladder (KUB) X-ray, computed tomography (CT), and fluoroscopy [5,6].

Finally, clinicians should consider the economic impact of urolithiasis management in terms of surgical interventions and diagnostic test imaging, particularly in recurrent stone formers (SFs). Unlike other pathologies, lithiasis affects the workforce in terms of age, indirectly affecting the disease’s cost. In the USA, it is estimated that the direct cost of lithiasis is about USD 4.5 billion, while the indirect cost is 750 million annually [7].

This review will delve into the latest research and clinical practices, providing a comprehensive overview of the current literature on recurrent stone formation and focusing on imaging modalities and endoscopic interventions.

## 2. Materials and Methods

### 2.1. Search Strategy

We systematically searched the Medline database (https://pubmed.ncbi.nlm.nih.gov, accessed on 1 April 2024) for articles regarding patients with recurrent urinary tract lithiasis. The limitations of using a single database for review were considered [8]. The keywords used were the following: “Nephrolith” OR “Kidney Calculus” OR “ROKS” OR “Kidney Stones” OR “Kidney Stone” OR “Urinary Calculi” OR “Renal Calculi” AND “Relapse” OR “Relapses” OR “Recurrences” OR “Recurrence” OR “Recurrent” OR “Repeat” OR “Retreatment” OR “Recrudescence” OR “Recrudescences” (all fields). Cmav and VT independently performed the retrieval on 1 April 2024. The references of the identified papers were also screened to determine further potential studies.

### 2.2. Selection Criteria

Our search was specifically tailored to identify studies on imaging strategies such as ultrasonography (US), CT, KUB X-ray, fluoroscopy, and pyelography. We also searched for relevant endoscopic treatment modalities such as ureteroscopy (URS), percutaneous nephrolithotomy (PNCL), retrograde intrarenal surgery (RIRS), or flexible URS for urinary tract lithiasis recurrence. We included original articles and case series published between 1 January 2014 and 1 April 2024, written in English. To maintain the relevance of our findings, we excluded articles on pregnant women and young people under 18, as well as surveys, reviews, guidelines, protocols, and case reports.

### 2.3. Data Extraction

For our data extraction, we focused on studies that reported on endoscopic surgical interventions and imaging modalities related to stone recurrence. We compiled all the relevant studies in a table, including the first author’s surname, publication year, population size and characteristics, follow-up time, the imaging modalities used, the urological interventions performed, and the primary outcomes reported. This comprehensive table serves as a valuable resource for our systematic search.

## 3. Results

Our comprehensive review encompasses a substantial body of research, incorporating 34 studies and a significant participant pool of 899,127 individuals [6,9,10,11,12,13,14,15,16,17,18,19,20,21,22,23,24,25,26,27,28,29,30,31,32,33,34,35,36,37,38,39,40,41].

### 3.1. Studies Selection

**Figure 1:**
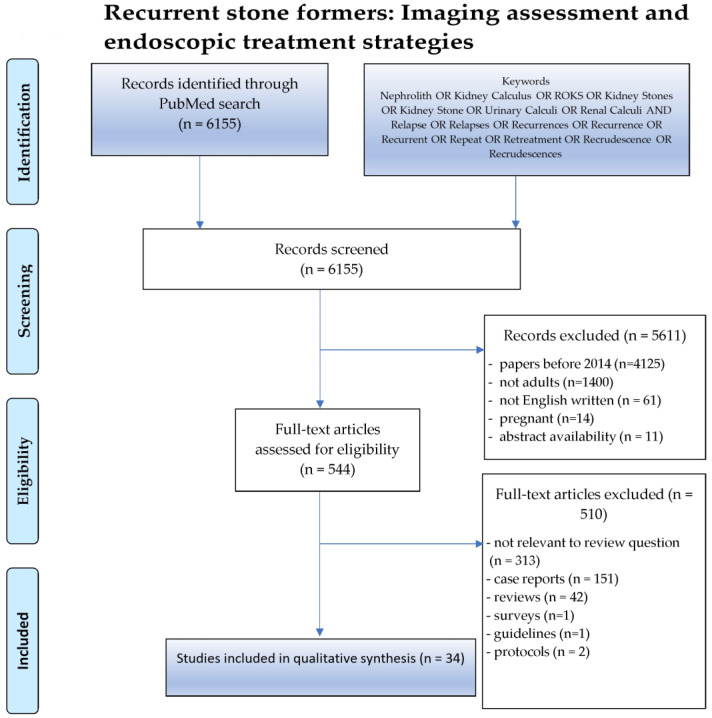
A flow diagram of the study selection process.

From an initial pool of 6155 results in the Medline database, we meticulously excluded 5611 studies that did not meet our rigorous criteria (articles before 2014, pregnant women, exclusively pediatric population, non-English, unavailable). The study by Kavoussi et al. was ultimately chosen due to its focus on an adult population [25]. Subsequently, 510 of the remaining 544 articles were excluded as they were reviews, protocols, surveys, case reports, or irrelevant to our research question. Finally, 34 studies were included in the qualitative synthesis, forming the robust foundation of our findings. Figure 1 provides a visual representation of this study’s flow diagram.

### 3.2. Imaging Modalities

Stone imaging techniques were reported in 27 of the 34 studies [6,9,10,11,12,13,14,16,18,19,20,21,22,23,24,25,26,28,29,30,31,33,34,35,36,40,41]. The imaging modality was not mentioned or clarified in the remaining nine studies, and the reference was not clarified [9,15,17,25,27,32,37,38,39]. Table 1 summarizes the studies referring to imaging modalities.

#### 3.2.1. CT

CT scanning was reported in a total of 21 studies. In most cases, no specific protocol was described beyond the standard practice of annual screening. Portis et al. re-examined patients one month after ureteral stent removal with a CT scan [31]. Hein et al. used low-dose CT only in symptomatic patients [21]. In contrast, Yamashita et al. used the preoperative imaging (non-contrast CT (NCCT)) of patients as mandatory for inclusion in their study [40]. Emiliani et al. recommended it only in patients with non-radiolucent stones [16], while Iremashvili et al. used it almost exclusively (in 92% of cases) in imaging in patients with urolithiasis [22]. Finally, CT was used as a predictive tool based on the Hounsfield unit (HU) for renal papillary, vertebral bone mineral, and abdominal aorta calcification [10,35,36].

#### 3.2.2. X-ray KUB

KUB X-ray was reported in nine studies. In Islam et al.’s study, KUB radiography was performed every six months after parathyroidectomy [23]. Ozgor et al. and Ito et al. also applied it twice a year [24,29,30]. In contrast, the percentage of patients in the Iremashvilli et al. study who underwent an X-ray was only 6.4% [22].

#### 3.2.3. US

Nine studies mentioned renal US, and three reported its use to monitor SFs every six months [24,29,30]. Sandhu et al. showed that US equals digital tomosynthesis (DT) in assessing kidney stones [34]. Zeng et al. compared the degree of hydronephrosis in cases of stone recurrence versus the first episode [41].

#### 3.2.4. DT

Two studies report using DT in the follow-up of SFs [12,34]. The study by Cabrera et al. highlights its effectiveness in monitoring patients with endonephric stones. While data on the ureter are limited, it generally seems to underperform compared to CT [12]. However, in the context of ureteral lithiasis, Sandu et al. demonstrated the clear superiority of DT over US [34].

### 3.3. Endoscopic Interventions

RIRS, URS, and PCNL are reported in 24 articles [6,11,14,15,16,17,18,19,20,21,22,24,25,26,28,29,30,31,32,37,38,39,40,41]. Table 2 summarizes the studies referring to surgical interventions in general.

#### 3.3.1. RIRS

Nine studies report RIRS as the only or one of the interventions for SFs [11,14,15,16,21,24,29,30,31]. In three, RIRS was the preferred surgical treatment [21,30,31]. Cohen et al. performed complete papillary mapping through RIRS to develop specific therapy strategies [15]. Ozgor et al. showed the method’s superiority over shockwave lithotripsy (SWL) for recurrent SFs [29]. Ito et al. found that residual fragments (RFs) > 4 mm after RIRS favor lithiasis recurrence [24]. Emiliani et al. showed the safety and efficacy of the surgery in elderly patients with a mean age of 81 [16].

#### 3.3.2. URS

URS was reported in 12 studies [6,19,20,22,25,28,31,32,37,38,39,40]. The study by Usawachintachit et al. showed that SFs undergoing URS due to bilateral stones are more likely to have repeat surgery [38]. Similarly, the large-scale study by Wang et al. among 556,217 participants showed that the HR for reoperation was 1.38 (1.31–1.45) [39].

#### 3.3.3. PCNL

Twelve studies included PCNL as a subject of analysis or as an intervention noted by the patient’s history [6,17,18,19,22,25,32,37,38,39,40,41]. Two studies by Evan et al. used PCNL to perform papillary mapping and biopsy without resulting in data that would help in the clinical management of recurrent SFs [17,18]. Ganesan et al. found that recurrent SFs with multiple sclerosis (MS) were more likely to undergo PCNL than those without (25 vs. 12%, *p* = 0.005) [19]. Usawachintachit et al. found that the recurrence rates of SFs undergoing PCNL did not differ between those with a unilateral stone and those with a bilateral stone [38]. Wang et al. showed that PCNL had the lowest HR relative to re-intervention (HR = 1.11, 1.06–1.18) [39].

## 4. Discussion

Urolithiasis is a very heterogeneous disease. There is a lack of comparative prospective studies between different imaging modalities of follow-up regarding the time intervals of the imaging needed and the modality of the initial surgical or conservative treatment. Most of the studies in this review were retrospective, with different aims, and a clear strategic plan for follow-up imaging cannot be determined. Moreover, they were not focused on the surgical treatment of recurrent SFs but rather on the metabolic profile and the medical treatment of the disease. An X-ray KUB and US are mostly suggested for follow-up, considering the stone composition, less radiation, financial burden, and the urologist’s preferences [42]. A recent systematic review trying to create an algorithm for follow-up for patients suffering from stone disease suggested that NCCT is preferred only in patients presenting with symptoms or undergoing an intervention [43]. A performed CT, however, according to others, was not an independent factor and did not contribute to the decision of reoperation during the follow-up [31]. Another study implied that the recurrence of the stone disease was milder, and the patients presenting with recurrence had less hydronephrosis than the first episode [41]. All this evidence seemed to be even more critical when CT scans were overused in patient follow-up since SFs were already exposed to significantly more radiation in comparison to non-stone patients or patients with inactive stone diseases from CT scans without even considering the intraoperative radiation. On average, active SFs received nearly ten times as many CTs as controls at three years (*p* < 0.001). The same study calculated that after one year of follow-up, an estimated 10.4% of operative SFs and 9.3% of nonoperative stone patients received 20–50 mSv in CT-related radiation, compared to 4.7% of inactive stone patients and 1.1% of controls (*p* < 0.001) [6]. Total stone burden and number were also independent factors that are associated not only with increased radiation during ureteroscopic management (stones with a diameter greater than 10 mm were associated with 37% higher radiation exposure compared to smaller stones (9.1 vs. 6.6 mGy, *p* < 0.001)), but also these patients are prone to undergoing NCCT and KUB 11.5 times more frequently compared to patients with low burden or having a single stone [20,28]. These facts could lead to the preference for the easily accessible and cost-efficient use of US as a first-line option for diagnosing stone disease in specific groups of patients. Ultrasound has a relatively low sensitivity of 45% but a high specificity of 94% for ureteral stones and the same sensitivity of 45% and a slightly lower specificity of 88% for renal stones [44]. A study also showed that patients initially assessed in the US did not have significant differences in hospital admissions, re-visits, pain, or high-risk adverse events without the CT field’s radiation exposure [45].

It is, therefore, of great importance to identify the patients at high risk of recurrence and attempt an individualized approach to treatment and follow-up so the morbidity of excessive radiation exposure and the number of reoperations can be reduced to the minimum amounts possible. Different studies included some factors and characteristics of either the patient or the stone that have some predictive value regarding the recurrence rate of the disease. The preoperative stone burden was one of them. However, a specific size or the number of stones cannot be defined within the studies, with the maximum stone size ranging from >7 mm to >2 cm being an independent and significant variable regarding the recurrence [24,25,28,30]. Stone composition is also an essential factor. Calcium oxalate stones were the most common and mixed with varying amounts of calcium phosphate. They comprised 80% of kidney stones [46]. However, other less common crystals, as seen from various studies, such as uric acid stones, calcium phosphate, struvite stones, ammonium acid urate stones, brushite, and cystine stones, seemed to be a predisposition for recurrent lithiasis and were more likely to re-emerge but also led to repeated surgical management [9,13,26,27,32,33,37]. A study also showed that symptomatic renal colic on the first episode with a stone in either the pelvis or lower calyx was a significant factor for reoperation with HR 1.66 (1.09–2.52). The same applied to any concurrent asymptomatic (non-obstructing) stone with HR 2.11 (1.36–3.26) [22]. Similar results regarding the recurrence and not the reoperation rate were found in another study, where concurrent asymptomatic or symptomatic stones in the pelvis or lower pole and a suspected previous renal colic were significant factors [33]. Some other variables regarding the patients such as younger age, male sex, various metabolic disorders (hyperlipidemia, obesity, etc.), family history, and previous interventions were all significant factors for recurrence [24,25,33,41]. In some specific occasions, such as multiple sclerosis, the time and method of bladder catheterization and the correct treatment of urinary tract infections were essential factors of stone recurrence [19]. The medullary sponge kidney alone was also an independent factor [18]. Finally, 25% of patients with primary hyperparathyroidism and nephrolithiasis who underwent successful parathyroidectomy presented with recurrent episodes independently of the normalization of serum calcium [23]. In those patients, it was, therefore, crucial to ensure the maximum stone-free (SFr) rate because even small residual fragments <1 mm, according to a study, increased the risk of a stone-related episode on the ipsilateral side (HR 2.823 CI (1.16, 6.85)) [21]. Another study also showed that residual fragments, especially those over 4 mm, seemed to increase the risk for stone-related episodes (HR 1.10 CI (1.05–1.17)) and that of future surgical re-interventions (HR 1.1 CI (1.05–1.17)) [24]. In one study, for stones from one to two cm, using flexible URS compared to SWL showed a reduced rate of residual fragments in favor of F-URS. This factor could reduce the recurrence and the reoperation ratio in high-risk patients [29]. Furthermore, pre-stenting patients with stones >1 cm were vital. Although it increased the cost of the initial operation by about six times and the amount of radiation by about 50%, it ultimately reduced the recurrence rate and contributed to reducing the overall financial and radiation burden [7,20]. Finally, according to a study, PCNL seems to have the lowest HR regarding re-intervention compared to SWL and URS. However, the study was retrospective, and the design was not a head-to-head comparison of the modalities [39].

One of the critical necessities regarding the individualized treatment of SFs is developing a reliable tool to predict the recurrence of lithiasis. Rule et al. introduced the recurrence of kidney stone (ROKS) prediction tool in 2014 [33]. This nomogram consisted of 13 questions, and the final result predicted recurrence at two, five years, and ten years. The initial nomogram addressed first-time SFs. In 2018, Vaughan et al. revised the nomogram by having 16 questions to allow for the prediction of the recurrence of lithiasis in patients with prior episodes [47]. Nevertheless, Iremashvili et al. showed that the ROKS nomogram had little clinical significance in their cohort among first-time SFs as they demonstrated limited calibration and discrimination (AUC = 0.655 and 0.605 for two years and five years recurrence, respectively), highlighting the need for a more accurate calculator [22]. On the other hand, the survey, including 261 experts in lithiasis, showed that their assessment of the recurrence of lithiasis differed from the ROKS nomogram [48]. Further disagreements existed between clinicians, and there was a tendency to avoid adopting calculators for individualized patient management [48]. Recently, the cohort of Kavoussi et al. showed relatively satisfactory performance of the ROKS nomogram on the recurrence of lithiasis at 2 and 5 years (ROC-AUC 0.67 and 0.63, respectively) [25]. In the future, a more precise calculator should be developed to estimate each patient’s recurrence risk and the potential radiation burden.

Although the literature search was systematic, several limitations exist. Most studies were retrospective and poorly designed. In addition, the data were not quantitatively analyzed. Furthermore, it needed to be more evident among the studies whether the recurrence was de novo or the progress of residual fragments, as there is no clear definition of SFR status. Some define it as no fragments postoperatively evaluated by the surgeon or radiologically. At the same time, the EAU guidelines’ consensus considers residual fragments < 4 mm as relatively insignificant as only one-third will eventually need a re-intervention [42]. Furthermore, the populations were remarkably heterogeneous concerning imaging modalities and the endoscopic management of recurrent SFs. Thus, the findings’ validity, reliability, and generalizability are significantly affected. We did not perform a risk of bias or a study quality assessment. Finally, a group of studies were conducted during the COVID-19 pandemic, which may have influenced their methodology and outcomes. Nevertheless, researchers failed to demonstrate significant differences in diagnostic and treatment strategies [49].

## 5. Conclusions

Urolithiasis is a complex and heterogeneous disease, and a specific algorithm for follow-up is not yet available. Patients with recurrent urinary tract lithiasis pose a unique challenge, both medically and economically. Urologists should consider the different aspects of the disease and individualize the imaging strategy and the required treatments/interventions for each patient separately. Pre-stenting is not to be avoided, especially in high-risk patients, and the modality chosen for treatment should aim at an SFr wherever possible. Clinicians also should not overuse CT scans in clinical practice and weigh the co-morbidity of radiation, especially in high-risk young patients, so that cumulative radiation exposure can be mediated. Treatment and follow-up plans should be holistic, aiming to apply appropriate imaging and surgical techniques. The lack of prospective comparative studies between different imaging modalities regarding the detection of clinically significant stones and the comparison of different interventional modalities at the initial treatment of recurrent SFs leaves plenty of space in this field of medicine for future research.

## Figures and Tables

**Table 1:** Studies on referenced imaging methods.

Reference	Year	Study	Population	Age	Gender	Imaging	Follow-Up	Outcomes
Ozgor et al. [30]	2014	Retrospective	44 patients (residual fragments < 5 mm)	Group 1, asymptomatic or stone-free (SFr) 44.1 + 13.4 Group 2, symptomatic 38 + 19.9	Group 1 (18 males, 11 females) Group 2 (9 males, 6 females)	KUB and abdominal US (twice per year). Abdominal CT (annually)	30.5 ± 8.809 months	Stone recurrence in 15 patients (34.1%). A total of 11 needed interventional therapy (5 RIRS, 2 SWL, 3 URS, and 1 PCNL). Size and number of residual stones were not associated with recurrence.
Rule et al. [33]	2014	Retrospective	2239 first-time symptomatic SFs	42 (32–54) 41 (31–51.0) in symptomatic recurrence patients	1399 males, 840 females	CT imaging without clarifying further details about the follow-up or radiation protocol	11.2 years	Surgery was weakly associated with a decreased risk of symptomatic recurrence but could not discriminate high- from low-risk patients. Prior incidental (asymptomatic) stone and ≥2 stones on imaging were risk factors for recurrence (unadjusted HRs = 1.53 (*p* = 0.004) and 1.72 (*p* < 0.001), respectively). Symptomatic recurrence rates at 2, 5, 10, and 15 years were 11%, 20%, 31%, and 39%, respectively.
Evan et al. [18]	2015	Retrospective	12 recurrent SFs with medullary sponge kidney	46.8 (36–66)	4 males, 8 females	KUB and CT	NA	The osteogenic theory was not supported. Urinary stasis could explain the pathophysiology of stone recurrence. Biopsy is not of value in the clinical management of patients with MSK.
Portis et al. [31]	2015	Retrospective	218 first-time SFs (burden < 1.5 cm)	51.5 ± 15	124 consecutive procedures in males and 102 in females	CT (1 month after stent removal)	4.1 years (interquartile range, 3.5–4.8)	A total of 19 patients (8.7%) needed repeat surgery (14 ureteroscopy and 5 PCNL). Total stone burden was associated with repeat surgery. Postoperative CT did not play a role in the need for reoperation. Cumulative repeat surgery rate was 5.8% and 8.6% at 1 and 5 years, respectively. A total of 26% of patients with fragments >2 to 4 mm and 46% of patients with fragments >4 mm needed reoperation.
Shavit et al. [36]	2015	Retrospective	111 (57 recurrent calcium SFs and 54 age- and sex-matched controls)	47 ± 14 (SFs), 47 ± 13 (controls)	63 males, 48 females	NCCT KUB (assessed the abdominal aortic calcification and vertebral bone mineral density)	NA	The abdominal aortic calcification severity score was significantly higher in SFs compared with the control group. The average vertebral bone mineral density was significantly lower in SFs.
Cabrera et al. [12]	2016	Retrospective	62 (the exact number of recurrent SFs was not clear)	42	NA	NCCT, DT	Mean time interval of 80.6 days between NCCT and DT.	DT could be used to follow-up patients with intrarenal lithiasis. Regarding the ureter, it is reported that NCCT identified 7 stones, while DT identified 1.
Hein et al. [21]	2016	Retrospective	85 SFr patients (included those with small fragments < 1 mm)	49.8 (17–80)	68 males, 17 females	No routine imaging in asymptomatic patients. Low-dose CT at first stone-related event	59 months (31–69)	Stone recurrence in 26 patients (30.1%). SWL in 6 patients, medical treatment in 5 patients, RIRS in 9, and multiple in 6. Residual fragments are an important predictor for ipsilateral stone recurrence both in high- and low-risk patients.
Shavit et al. [35]	2016	Prospective	111 (57 recurrent nephrolithiasis and 54 age- and sex-matched controls)	47 ± 14 (nephrolithiasis) 47 ± 13 (without lithiasis)	63 males, 48 females	NCCT	NA	Increased HU papillary density in the recurrent SFs regardless of whether they are hypercalciuric or non-hypercalciuric.
Ganesan et al. [19]	2017	Retrospective	181 recurrent SFs (61 patients with MS compared with 120 matched controls)	53.7 (47.0–61.3, No MS) 53.2 (47.0–58.1, MS)	57 males, 124 females	CT (when available)	3.9 (1.5–7.7) years	MS patients more likely to have undergone a PCNL (25% vs. 12%, *p* = 0.005) with a higher proportion of struvite and calcium phosphate and stones (8% vs. 3%, *p* = 0.03 and 42% vs. 15%, *p* < 0.001, respectively). Less likely to have calcium oxalate monohydrate stones (39% vs. 64%, *p* < 0.001).
Bhojani et al. [11]	2018	Prospective	11 known previous calcium oxalate SFs	48 (21–74)	7 males, 4 females	NCCT (helical)	NA	Non-contrast helical CT underestimates the total number of kidney stones compared to endoscopy (9.2 ± 6.1 vs. 5.9 ± 4.1, *p* < *0*.004) but did not differ in total stone burden.
Hadjipavlou et al. [20]	2018	Retrospective	302 SFs with single calculus until stone clearance	56 (21–92)	195 males, 107 females	NCCT KUB all patients, while 85 (29%) also had at least one X-ray	NA	Large stone burden, proximal stone location, and truncal obesity were associated with higher ionizing radiation during URS. Pre-stenting was associated with over 50% of the radiation delivered during URS (4.13 vs. 7.54 mGy, respectively).
Ozgor et al. [29]	2018	Retrospective	113 and 128 patients with lower pole renal stone underwent SWL and RIRS, respectively	45.9 ± 14.7 (RIRS) 48.6 ± 14.9 (SWL)	128 males, 113 females	US and X-ray KUB twice per year. CT annually	34.1 ± 13.2 months (RIRS) 32.4 ± 8.5 months (SWL)	Stone recurrence was more common in SWL (28 vs. 17 patients, *p* = 0.009). No differences for the recurrent stone size and recurrence period.
Sandhu et al. [34]	2018	Prospective	66 (including recurrent SFs)	44.2 (19–73)	36 males, 30 females	US, DT, and NCCT	ΝA	DT is not superior to the US in the detection of kidney stones but still performs better in the identification of ureteral stones.
Yamashita et al. [40]	2018	Retrospective	300 patients with upper urinary tract stones (148 recurrent, 152 first-time)	59 (49–69)	208 males, 98 females	Pretreatment NCCT	NA	The visceral fat ratio was greater in patients with recurrent lithiasis compared to first-time formers despite the fact that there were no statistically significant differences in BMI and waist circumference.
Arda et al. [10]	2019	Retrospective	280 (98 recurrent SFs, 88 primary and 94 age-matched control participants)	34 (21–60, primary) 37 (21–67, recurrent)	NA	NCCT (helical)	NA	Higher papillae HU could predict stone recurrence.
Canales et al. [13]	2019	Retrospective	53 uric acid SFs	61.4 ± 11.7	35 males, 18 females	Routinely followed at 6-month intervals with CT	25 months (15.8–47.5)	A total of 32% of patients (17) had stone recurrence, and 13% (7) required surgical intervention. Mean time interval to stone recurrence was 16.8 ± 15.3 months. The 32% of patients had CT-documented stone recurrences over the 2-year interval regardless of therapy.
Dai et al. [6]	2019	Retrospective	327,516 (active stone, 112,140 were ted operatively and 215,376 were managed nonoperatively) 175,228 inactive stone patients and 502,744 age- and gender-matched controls	18–65 (divided into 4 groups, 18–39, 40–49, 50–59 and 60–65)	552,052 males, 453,436 females	Standard dose CT	3 years of continuous follow-up	The 3-year cumulative CT radiation (mean) was 28.3 ± 28.5 mSv for operative patients and 22.0 ± 24.4 mSv for nonoperative patients. For inactive stone patients and controls, exposure was 14.9 ± 19.3 mSv and 2.4 ± 10.0 mSv, respectively. Surgical-treated patients receive 9 to 12 times more CT scans than age- and sex-matched controls during the 3-year follow-up. Also, more than 10% of them exceeded occupational risk thresholds in the first year without accounting for exposure during surgical procedures.
Iremashvili et al. [22]	2019	Retrospective	498 SFs	53.6 (18.1–89.6)	260 males, 238 females	CT (92%), US (1.6%), X-ray (6.4%)	4.8 years (mean 4.6, IQR 3.1–6.1)	A total of 88 patients (17.7%) were symptomatic recurrent SFs at 5-year follow-up, requiring surgical treatment. This percentage increased to 25% by 8 years of follow-up. Symptomatic stone (renal pelvis or lower renal pole) and any concurrent non-obstructing, asymptomatic stone associated with the risk of repeat surgery (HR 1.66 (1.09–2.52), *p* = 0.018 and 2.11 (1.36–3.26) *p* = 0.001, respectively)
Zeng et al. [41]	2019	Retrospective	146 recurrent SFs (of the 3985–3.7%)	Males’ mean 39.4 ± 15.8 Females’ mean age 41.5 ± 18.5	100 males, 48 females	Ultrasound assessment of severity of hydronephrosis	4 years	Minimally invasive methods were mainly used to treat the first stone episode. The main symptom of recurrence was associated with infection (renal colic at first episode). Recurrences of lithiasis occur mainly with a lesser degree of hydronephrosis.
Abbassene et al. [9]	2020	Retrospective	1104 SFs	45.3 ± 13.9	727 males, 377 females	Imaging modalities are not clarified	Over 7 years	A total of 51.1% of patients had stone recurrence (prevalence of males). Cystine was the most recurrent stone (27.3% consanguinity).
Islam et al. [23]	2020	Retrospective	69 SFs with primary hyperparathyroidism (Parathyroidectomy)	57 ± 14	31 males, 38 females	X-ray KUB every 6 months after parathyroidectomy	4.0 ± 2.9 years	A total of 23% of patients (16 of 69) had stone recurrence after parathyroidectomy, and 88% (14) needed surgical intervention. Younger patients are high-risk for stone recurrence.
Ito et al. [24]	2021	Retrospective	664 SFs	60.0 ± 12.7	418 males, 246 females	X-ray KUB and US at first month, followed by once every 6 months.	31.1 months	A total of 15.5% of patients (103) experienced surgical intervention (40.8% URS, 56.3% SWL, and 2.9% PCNL). Stone burden ≥ 20 mm and RF ≥ 4 mm were predictive factors for stone recurrence.
Li et al. [26]	2021	Retrospective	1051 SFs	59.1 ± 15.1	555 males, 496 females	US, X-ray KUB and CT with no standardized plan	4.7 ± 2.5 years	A total 26.7% of patients required repeat surgery. Cystine and brushite SFs had the highest and the second-highest risk for surgical recurrence, respectively.
Emiliani et al. [16]	2021	Retrospective	173 SFs (78 for group 1, age < 80 and 95 for group 2, age ≥ 80	group 1 → 44 (27–79) group 2 → 81 (80–94)	group 1 → 40 males, 38 females group 2 → 45 males, 50 females	US and NCCT (for non-radio-opaque stones) at 1 year	Not clarified	Recurrence rate did not differ between group 1 and group 2 (4.3% vs. 5.6%, *p* = 0.730, respectively). Elderly patients were controlled with longer operative time and hospitalization than younger patients. There was no difference regarding complications.
Mancuso et al. [28]	2022	Retrospective	79 patients with multiple same-sided ureteric stones and 101 with single ureteric stone	57 ± 13 (multiple) 49 ± 15 (single)	122 males, 58 females	US, X-ray KUB, and NCCT	Not clarified	Patients with multiple stones were more likely to be recurrent SFs and formed a higher proportion of non-calcium oxalate stones. Also, they were more likely to have other procedures (except URS) such as PCNL. They underwent NCCT and KUB 11.5 times more frequently compared with single ureteric SFs.
Kavoussi et al. [25]	2023	Retrospective	200 patients (100 without stone recurrence and 100 with recurrence)	49 ± 15 (overall) 47 ± 15 (recurrence) 52 ± 6 (non-recurrence)	101 males, 99 females	at least yearly imaging	96 ± 38 months	Time to stone recurrence was 29 ± 32 months. Calcium oxalate monohydrate stone, family history of stone disease and a stone > 6 mm in diameter associated with risk of recurrent lithiasis. The surgical method was not associated with stone recurrence.
Chai et al. [14]	2023	Retrospective	6579 SFs (3112 pre-stenting 3467 no stenting)	49.34 ± 15.59 (overall) 51.44 ± 16.01 (pre-stenting) 47.47 ± 14.94 (no stenting)	4346 males, 2233 females	CT, US, KUB X-rays with no standardized settings	NA	Recurrent SFs had higher overall complications and residual fragments.

Computed tomography (CT), digital tomosynthesis (DT), Hounsfield unit (HU), interquartile range (IQR), kidney–ureter–bladder (KUB), multiple sclerosis (MS), medullary sponge kidney (MSK), non-contrast CT (NCCT), percutaneous nephrolithotomy (PCNL), residual fragment (RF), retrograde intrarenal surgery (RIRS), shockwave lithotripsy (SWL), stone formers (SFs), stone-free (SFr), ureteroscopy (URS), ultrasonography (US).

**Table 2:** Studies on referenced endoscopic procedures.

Reference	Year	Study	Population	Age	Gender	Interventions	Follow-Up	Outcomes
Ozgor et al. [30]	2014	Retrospective	44 patients (residual fragments < 5 mm)	Group 1, asymptomatic or SFr 44.1 + 13.4 Group 2, symptomatic 38 + 19.9	Group 1 (18 males, 11 females) Group 2 (9 males, 6 females)	RIRS	30.5 ± 8.809 months	Stone recurrence in 15 patients (34.1%). A total of 11 needed interventional therapy (5 RIRS, 2 SWL, 3 URS, and 1 PCNL) Size and number of residual stones were not associated with recurrence.
Rule et al. [33]	2014	Retrospective	2239 first-time symptomatic SFs	42 (32–54) 41 (31, 51.0) in symptomatic recurrence patients	1399 males, 840 females	Surgery documented in 33% of patients. No surgical intervention was included in the final nomogram.	11.2 years	Surgery was weakly associated with a decreased risk of symptomatic recurrence but could not discriminate high- from low-risk patients. Prior incidental (asymptomatic) stone and ≥2 stones on imaging were risk factors for recurrence (unadjusted HRs = 1.53 (*p* = 0.004) and 1.72 (*p* < 0.001), respectively). Symptomatic recurrence rates at 2, 5, 10, and 15 years were 11%, 20%, 31%, and 39%, respectively.
Evan et al. [18]	2015	Retrospective	12 recurrent SFs with medullary sponge kidney	46.8 (36–66)	4 males, 8 females	PCNL with papillary biopsy	NA	The osteogenic theory was not supported. Urinary stasis could explain the pathophysiology of stone recurrence. Biopsy is not of value in the clinical management of patients with MSK.
Portis et al. [31]	2015	Retrospective	218 first time SFs (burden < 1.5 cm)	51.5 ± 15	124 consecutive procedures in males and 102 in females	Rigid ureteroscopy (below the iliac vessels) flexible ureteroscopy (in the upper tract)	4.1 years (interquartile range, 3.5–4.8)	A total of 19 patients (8.7%) needed repeat surgery (14 ureteroscopy and 5 PCNL). Total stone burden was associated with repeat surgery. Postoperative CT did not play a role in the need for reoperation. Cumulative repeat surgery rate was 5.8% and 8.6% at 1 and 5 years, respectively. A total of 26% of patients with fragments >2 to 4 mm and 46% of patients with fragments >4 mm needed reoperation.
Hein et al. [21]	2016	Retrospective	85 SFr patients (included those with small fragments < 1 mm)	49.8 (17–80)	68 males, 17 females	RIRS	59 months (31–69)	Stone recurrence in 26 patients (30.1%). SWL in 6 patients, medical treatment in 5 patients, RIRS in 9, and multiple in 6. Residual fragments are an important predictor for ipsilateral stone recurrence both in high- and low-risk patients.
Cohen et al. [15]	2017	Prospective	13 patients (100% recurrent SFs, center 1) 63 patients (54.2% recurrent SFs, center 2)	46 (42–52, center 1) 51 (41–63, center 2)	42 males, 34 females	RIRS with complete papillary mapping	NA	Recurrent SFs had higher total scores (papillary mapping). The scoring system during endoscopy may have clinical usefulness for adapting specific treatment strategies.
Ganesan et al. [19]	2017	Retrospective	181 recurrent s SFs (61 patients with MS compared with 120 matched controls)	53.7 (47.0–61.3, No MS) 53.2 (47.0–58.1, MS)	53.7 (47.0–61.3, No MS) 53.2 (47.0–58.1, MS)	URS, PCNL, SWL, Cystolithopaxy	3.9 (1.5–7.7) years	MS patients more likely to have undergone a PCNL (25% vs. 12%, *p* = 0.005) with a higher proportion of struvite and calcium phosphate and stones (8% vs. 3%, *p* = 0.03 and 42% vs. 15%, *p* < 0.001, respectively). Less likely to have calcium oxalate monohydrate stones (39% vs. 64%, *p* < 0.001).
Lomas et al. [27]	2017	Retrospective	89 patients with ammonium acid urate stones	55 (39.5–70.5) at stone formation	38 males, 17 females	83% required surgical intervention (not clarified the method)	4.9 years (IQR 1.8–8.5),	A total of 19 patients (21%) had stone recurrence with a median time to recurrence of 22 months (IQR 10.5–42).
Rivera et al. [32]	2017	Retrospective	20 brushite SFs matched with 60 calcium oxalate SFs	48 (38–58, brushite) 47 (40–56, calcium oxalate)	57 males, 23 females	Prior stone surgery as SWL, PCNL or URS in 60% and 33% of brushite and calcium oxalate SFs, respectively	12.2 years (10.0–17.4, brushite) 13.5 years (10.4–17.0, calcium oxalate)	Brushite SFs had a higher incidence of prior stones as well as prior surgical intervention. Also, they had a higher stone recurrence rate (80% vs. 42%, *p* = 0.003) without changing in the CKD stage.
Streeper et al. [37]	2017	Retrospective	12 cystine matched with 12 non-cystine recurrent SFs	50.6 ± 16.7 (cystine) 53.5 ± 16.8 (non cystine)	6 males, 18 females	URS, PCNL, SWL	5 years	Cystine SFs had a greater number of surgical interventions compared to non-cystine formers (8.5 ± 9.1 vs. 2.9 ± 3.1). 62% of cystine formers underwent PCNL, whereas the percentage of non-cystine patients was 15%. Similarly, for ureteroscopy, the percentages were 100% vs. 54%.
Bhojani et al. [11]	2018	Prospective	11 known previous calcium oxalate SFs	48 (21–74)	7 males, 4 females	RIRS	NA	NCCT (helical) underestimated the total number of kidney stones compared to endoscopy (9.2 ± 6.1 vs. 5.9 ± 4.1, *p* < 0.004) but did not differ in total stone burden.
Hadjipavlou et al. [20]	2018	Retrospective	302 SFs with single calculus until stone clearance	56 (21–92)	195 males, 107 females	URS	NA	Large stone burden, proximal stone location, and truncal obesity were associated with higher ionizing radiation during URS. Pre-stenting was associated with over 50% of the radiation delivered during URS (4.13 vs. 7.54 mGy, respectively).
Ozgor et al. [29]	2018	Retrospective	113 and 128 patients with lower pole renal stone underwent SWL and RIRS, respectively	45.9 ± 14.7 (RIRS) 48.6 ± 14.9 (SWL)	128 males, 113 females	RIRS	34.1 ± 13.2 months (RIRS) 32.4 ± 8.5 months (SWL)	Stone recurrence was more common in SWL (28 vs. 17 patients, *p* = 0.009). No differences for the recurrent stone size and recurrence period.
Usawachintachit et al. [38]	2018	Retrospective	42 recurrent cystine stone patients	45.5 (IQR: 28–63)	20 males, 22 females	PCNL, URS, SWL, open surgery	8.8 years (0.9–13.6)	A total of 2/3 of patients form bilateral stones which correlated with higher median number of lifetime URS compared with unilateral SFs (2 vs. 1 session, *p* < 0.05). There was no significant difference for PCNL (*p* = 0.55).
Yamashita et al. [40]	2018	Retrospective	300 patients with upper urinary tract stones (148 recurrent, 152 first-time)	59 (49–69)	208 males, 98 females	PCNL, URS, SWL	NA	The visceral fat ratio was greater in patients with recurrent lithiasis compared to first-time formers despite the fact that there were no statistically significant differences in BMI and waist circumference.
Canales et al. [13]	2019	Retrospective	53 uric acid SFs	61.4 ± 11.7	35 males, 18 females	Not clarified	25 months (15.8–47.5)	A total of 32% of patients (17) had stone recurrence, and 13% (7) required surgical intervention. Mean time interval to stone recurrence was 16.8 ± 15.3 months. The 32% of patients had CT-documented stone recurrences over the 2-year interval regardless of therapy.
Dai et al. [6]	2019	Retrospective	327,516 (active stone, 112,140 were ted operatively and 215,376 were managed nonoperatively) 175,228 inactive stone patients and 502,744 age- and gender-matched controls	18–65 (divided into 4 groups, 18–39, 40–49, 50–59 and 60–65)	552,052 males, 453,436 females	PCNL, URS, SWL	3 years of continuous follow-up	The 3-year cumulative CT radiation (mean) was 28.3 ± 28.5 mSv for operative patients and 22.0 ± 24.4 mSv for nonoperative patients. For inactive stone patients and controls, exposure was 14.9 ± 19.3 mSv and 2.4 ± 10.0 mSv, respectively. Surgical-treated patients receive 9 to 12 times more CT scans than age- and sex-matched controls during the 3-year follow-up. Also, more than 10% of them exceeded occupational risk thresholds in the first year without accounting for exposure during surgical procedures.
Iremashvili et al. [22]	2019	Retrospective	498 SFs	53.6 (18.1–89.6)	260 males, 238 females	PCNL (12.2%), Ureteroscopy (83.5%), SWL (4.2%)	4.8 years (mean 4.6, IQR 3.1–6.1)	A total of 88 patients (17.7%) were symptomatic recurrent SFs at 5-year follow-up, requiring surgical treatment. This percentage increased to 25% by 8 years of follow-up. Symptomatic stone (renal pelvis or lower renal pole) and any concurrent non-obstructing, asymptomatic stone associated with the risk of repeat surgery (HR 1.66 (1.09–2.52), *p* = 0.018 and 2.11 (1.36–3.26) *p* = 0.001, respectively).
Zeng et al. [41]	2019	Retrospective	146 recurrent SFs (of the 3985–3.7%)	Males’ mean 39.4 ± 15.8 Females’ mean age 41.5 ± 18.5	100 males, 48 females	PCNL (65 cases) SWL (22 cases), super-mini PCNL (20 cases), open surgery (four cases) and ureteroscopy (one case).	4 years	Minimally invasive methods were mainly used to treat the first stone episode. The main symptom of recurrence was associated with infection (renal colic at first episode). Recurrences of lithiasis occur mainly with a lesser degree of hydronephrosis.
Abbassene et al. [9]	2020	Retrospective	1104 SFs	45.3 ± 13.9	727 males, 377 females	Conventional surgery (19.7%) endourology or SWL (15.5%)	Over 7 years	A total of 51.1% of patients had stone recurrence (prevalence of males). Cystine was the most recurrent stone (27.3% consanguinity).
Evan et al. [17]	2020	Retrospective	8 uric acid SFs (6 recurrent)	45.5 (37–67)	8 males	PCNL (Papillary mapping and biopsy)	NA	Renal papillae plaques and plugging did not correlate with patients’ prior episodes of symptomatic lithiasis (very small number of cases).
Islam et al. [23]	2020	Retrospective	69 SFs with primary hyperparathyroidism (Parathyroidectomy)	57 ± 14	31 males, 38 females	Not clarified	4.0 ± 2.9 years	A total of 23% of patients (16 of 69) had stone recurrence after parathyroidectomy, and 88% (14) needed surgical intervention. Younger patients are high-risk for stone recurrence.
Ito et al. [24]	2021	Retrospective	664 SFs	60.0 ± 12.7	418 males, 246 females	RIRS	31.1 months	A total of 15.5% of patients (103) experienced surgical intervention (40.8% URS, 56.3% SWL, and 2.9% PCNL). Stone burden ≥ 20 mm, and RF ≥ 4 mm were predictive factors for stone recurrence.
Li et al. [26]	2021	Retrospective	1051 SFs	59.1 ± 15.1	555 males, 496 females	URS, PCNL, SWL	4.7 ± 2.5	A total of 26.7% of patients required repeat surgery. Cystine and brushite SFs had the highest and the second-highest risk for surgical recurrence, respectively.
Emiliani et al. [16]	2021	Retrospective	173 SFs (78 for group 1, age < 80 and 95 for group 2, age≥ 80	group 1 → 44 (27–79) group 2 → 81 (80–94)	group 1 → 40 males, 38 females group 2 → 45 males, 50 females	RIRS	Not clarified	Recurrence rate did not differ between group 1 and group 2 (4.3% vs. 5.6%, *p* = 0.730, respectively). Elderly patients were controlled with longer operative time and hospitalization than younger patients. There was no difference regarding complications.
Mancuso et al. [28]	2022	Retrospective	79 patients with multiple same-sided ureteric stones and 101 with single ureteric stone	57 ± 13 (multiple) 49 ± 15 (single)	122 males, 58 females	SWL, URS	Not clarified	Patients with multiple stones were more likely to be recurrent SFs and formed a higher proportion of non-calcium oxalate stones. Also, they were more likely to have other procedures (except URS) such as PCNL. They underwent NCCT and KUB 11.5 times more frequently compared with single ureteric SFs.
Kavoussi et al. [25]	2023	Retrospective	200 patients (100 without stone recurrence and 100 with recurrence)	49 ± 15 (overall) 47 ± 15 (recurrence) 52 ± 6 (non-recurrence)	101 males, 99 females	URS, PCNL, SWL	96 ± 38 months	Time to stone recurrence was 29 ± 32 months. Calcium oxalate monohydrate stone, family history of stone disease, and a stone > 6 mm in diameter were associated with the risk of recurrent lithiasis. The surgical method was not associated with stone recurrence.
Chai et al. [14]	2023	Retrospective	6579 (3112 pre-stenting 3467 no stenting)	49.34 ± 15.59 (overall) 51.44 ± 16.01 (pre-stenting) 47.47 ± 14.94 (no stenting)	4346 males, 2233 females	RIRS	NA	Recurrent SFs had higher overall complications and residual fragments.
Wang et al. [39]	2024	Retrospective	556,217 patients with upper urinary tract stone	49.9 ± 13.1	356,532 males, 199,685 females	Open surgery (8.6%), SWL (8.4%), URS (53.4%), and PCNL (29.6%)	2.7 years (IQR 1.5–4.0)	A total of 23,012 patients (4.1%) underwent a second surgical intervention (incidence rate of 14.9 per 1000 person-years). Hazard ratios for SWL, ureteroscopic lithotripsy, and PCNL were 1.59 (1.49–1.70), 1.38 (1.31–1.45), and 1.11 (1.06–1.18), respectively.

## Data Availability

Not applicable.

## References

[1] Stamatelou K., Goldfarb D.S. (2023). Epidemiology of Kidney Stones. Healthcare.

[2] Sorokin I., Mamoulakis C., Miyazawa K., Rodgers A., Talati J., Lotan Y. (2017). Epidemiology of stone disease across the world. World J. Urol..

[3] Tamborino F., Cicchetti R., Mascitti M., Litterio G., Orsini A., Ferretti S., Basconi M., De Palma A., Ferro M., Marchioni M. (2024). Pathophysiology and Main Molecular Mechanisms of Urinary Stone Formation and Recurrence. Int. J. Mol. Sci..

[4] D’Ambrosio V., Ferraro P.M., Lombardi G., Friso S., Gambaro G. (2022). Unravelling the Complex Relationship between Diet and Nephrolithiasis: The Role of Nutrigenomics and Nutrigenetics. Nutrients.

[5] Ferrandino M.N., Bagrodia A., Pierre S.A., Scales C.D., Rampersaud E., Pearle M.S., Preminger G.M. (2009). Radiation exposure in the acute and short-term management of urolithiasis at 2 academic centers. J. Urol..

[6] Dai J.C., Chang H.C., Holt S.K., Harper J.D. (2019). National Trends in CT Utilization and Estimated CT-related Radiation Exposure in the Evaluation and Follow-up of Stone Patients. Urology.

[7] Hyams E.S., Matlaga B.R. (2014). Economic impact of urinary stones. Transl. Androl. Urol..

[8] Falagas M.E., Pitsouni E.I., Malietzis G.A., Pappas G. (2008). Comparison of PubMed, Scopus, Web of Science, and Google Scholar: Strengths and weaknesses. FASEB J. Off. Publ. Fed. Am. Soc. Exp. Biol..

[9] Abbassene F., Maizia A., Messaoudi N., Bendahmane L., Boukharouba H., Daudon M., Addou A. (2020). Adult urolithiasis in Western Algeria: A study of 1104 cases. Tunis. Med..

[10] Arda E., Cakıroglu B., Akdeniz E., Yuksel I., Cetin G., Hilmi Aksoy S. (2019). Comparison of Turkish Primary, Recurrent, and Non Stone-Forming Patients Using Hounsfield Unit Measurements: How Useful Is It?. Curr. Urol..

[11] Bhojani N., Paonessa J.E., El Tayeb M.M., Williams J.C., Hameed T.A., Lingeman J.E. (2018). Sensitivity of Noncontrast Computed Tomography for Small Renal Calculi With Endoscopy as the Gold Standard. Urology.

[12] Cabrera F.J., Kaplan A.G., Youssef R.F., Tsivian M., Shin R.H., Scales C.D., Preminger G.M., Lipkin M.E. (2016). Digital Tomosynthesis: A Viable Alternative to Noncontrast Computed Tomography for the Follow-Up of Nephrolithiasis?. J. Endourol..

[13] Canales B.K., Sharma N., Yuzhakov S.V., Bozorgmehri S., Otto B.J., Bird V.G. (2019). Long-term Recurrence Rates in Uric Acid Stone Formers With or Without Medical Management. Urology.

[14] Chai C.A., Teoh Y.C., Tailly T., Emiliani E., Inoue T., Tanidir Y., Gadzhiev N., Bin Hamri S., Ong W.L., Shrestha A. (2023). Influence of pre-stenting on RIRS outcomes. Inferences from patients of the Global Multicentre Flexible Ureteroscopy Outcome Registry (FLEXOR). Minerva Urol. Nephrol..

[15] Cohen A.J., Borofsky M.S., Anderson B.B., Dauw C.A., Gillen D.L., Gerber G.S., Worcester E.M., Coe F.L., Lingeman J.E. (2017). Endoscopic Evidence That Randall’s Plaque is Associated with Surface Erosion of the Renal Papilla. J. Endourol..

[16] Emiliani E., Piccirilli A., Cepeda-Delgado M., Kanashiro A.K., Mantilla D., Amaya C.A., Sanchez-Martin F.M., Millan-Rodriguez F., Territo A., Amón-Sesmero J.H. (2021). Flexible ureteroscopy in extreme elderly patients (80 years of age and older) is feasible and safe. World J. Urol..

[17] Evan A.P., Coe F.L., Worcester E.M., Williams J.C., Heiman J., Bledsoe S., Sommer A., Philips C.L., Lingeman J.E. (2020). Discrepancy Between Stone and Tissue Mineral Type in Patients with Idiopathic Uric Acid Stones. J. Endourol..

[18] Evan A.P., Worcester E.M., Williams J.C., Sommer A.J., Lingeman J.E., Phillips C.L., Coe F.L. (2015). Biopsy proven medullary sponge kidney: Clinical findings, histopathology, and role of osteogenesis in stone and plaque formation. Anat. Rec..

[19] Ganesan V., Chen W.M., Jain R., De S., Monga M. (2017). Multiple sclerosis and nephrolithiasis: A matched-case comparative study. BJU Int..

[20] Hadjipavlou M., Lam V., Seth J., Anjum F., Sriprasad S. (2018). Radiation Exposure during Ureterorenoscopy and Laser Lithotripsy: An Analysis of Stone Characteristics. Urol. Int..

[21] Hein S., Miernik A., Wilhelm K., Schlager D., Schoeb D.S., Adams F., Vach W., Schoenthaler M. (2016). Endoscopically Determined Stone Clearance Predicts Disease Recurrence Within 5 Years After Retrograde Intrarenal Surgery. J. Endourol..

[22] Iremashvili V., Li S., Penniston K.L., Best S.L., Hedican S.P., Nakada S.Y. (2019). External Validation of the Recurrence of Kidney Stone Nomogram in a Surgical Cohort. J. Endourol..

[23] Islam A.K., Holt S., Reisch J., Nwariaku F., Antonelli J., Maalouf N.M. (2020). What Predicts Recurrent Kidney Stone after Parathyroidectomy in Patients with Primary Hyperparathyroidism?. J. Am. Coll. Surg..

[24] Ito K., Takahashi T., Somiya S., Kanno T., Higashi Y., Yamada H. (2021). Predictors of Repeat Surgery and Stone-related Events After Flexible Ureteroscopy for Renal Stones. Urology.

[25] Kavoussi N.L., Da Silva A., Floyd C., McCoy A., Koyama T., Hsi R.S. (2023). Feasibility of stone recurrence risk stratification using the recurrence of kidney stone (ROKS) nomogram. Urolithiasis.

[26] Li S., Iremashvili V., Vernez S.L., Penniston K.L., Jhagroo R.A., Best S.L., Hedican S.P., Nakada S.Y. (2021). Effect of stone composition on surgical stone recurrence: Single center longitudinal analysis. Can. J. Urol..

[27] Lomas D.J., Jaeger C.D., Krambeck A.E. (2017). Profile of the Ammonium Acid Urate Stone Former Based on a Large Contemporary Cohort. Urology.

[28] Mancuso M., Lavoie C., Assmus M., De S. (2022). Characterizing patients with multiple same-sided ureteric stones. World J. Urol..

[29] Ozgor F., Sahan M., Yanaral F., Savun M., Sarilar O. (2018). Flexible ureterorenoscopy is associated with less stone recurrence rates over Shockwave lithotripsy in the management of 10–20 millimeter lower pole renal stone: Medium follow-up results. Int. Braz. J. Urol..

[30] Ozgor F., Simsek A., Binbay M., Akman T., Kucuktopcu O., Sarilar O., Muslumanoglu A.Y., Berberoglu Y. (2014). Clinically insignificant residual fragments after flexible ureterorenoscopy: Medium-term follow-up results. Urolithiasis.

[31] Portis A.J., Laliberte M.A., Heinisch A. (2015). Repeat Surgery After Ureteroscopic Laser Lithotripsy With Attempted Complete Extraction of Fragments: Long-term Follow-up. Urology.

[32] Rivera M., Jaeger C., Yelfimov D., Krambeck A.E. (2017). Risk of Chronic Kidney Disease in Brushite Stone Formers Compared With Idiopathic Calcium Oxalate Stone Formers. Urology.

[33] Rule A.D., Lieske J.C., Li X., Melton L.J., Krambeck A.E., Bergstralh E.J. (2014). The ROKS nomogram for predicting a second symptomatic stone episode. J. Am. Soc. Nephrol..

[34] Sandhu M.S., Gulati A., Saritha J., Nayak B. (2018). Urolithiasis: Comparison of diagnostic performance of digital tomosynthesis and ultrasound. Which one to choose and when?. Eur. J. Radiol..

[35] Shavit L., Girfoglio D., Kirkham A., Allen D., Ferraro P.M., Moochhala S., Unwin R. (2016). Increased renal papillary density in kidney stone formers detectable by CT scan is a potential marker of stone risk, but is unrelated to underlying hypercalciuria. Urolithiasis.

[36] Shavit L., Girfoglio D., Vijay V., Goldsmith D., Ferraro P.M., Moochhala S.H., Unwin R. (2015). Vascular calcification and bone mineral density in recurrent kidney stone formers. Clin. J. Am. Soc. Nephrol..

[37] Streeper N.M., Wertheim M.L., Nakada S.Y., Penniston K.L. (2017). Cystine Stone Formers Have Impaired Health-Related Quality of Life Compared with Noncystine Stone Formers: A Case-Referent Study Piloting the Wisconsin Stone Quality of Life Questionnaire Among Patients with Cystine Stones. J. Endourol..

[38] Usawachintachit M., Sherer B., Hudnall M., Tzou D.T., Taguchi K., Hsi R.S., Stoller M., Chi T. (2018). Clinical Outcomes for Cystinuria Patients with Unilateral Versus Bilateral Cystine Stone Disease. J. Endourol..

[39] Wang Q., Wang Y., Yang C., Wang J., Zhang X.C., Zhang L., Zhao M.H. (2024). Surgical procedure and recurrence of upper urinary tract stone: A national-wide study based on hospitalized patients. World J. Urol..

[40] Yamashita S., Iguchi T., Nishizawa S., Iba A., Kohjimoto Y., Hara I. (2018). Recurrent stone-forming patients have high visceral fat ratio based on computed tomography images compared to first-time stone-forming patients. Int. J. Urol..

[41] Zeng J., Wang S., Zhong L., Huang Z., Zeng Y., Zheng D., Zou W., Lai H. (2019). A Retrospective Study of Kidney Stone Recurrence in Adults. J. Clin. Med. Res..

[42] Tzelves L., Geraghty R., Lombardo R., Davis N.F., Petřík A., Neisius A., Gambaro G., Türk C., Thomas K., Somani B. (2023). Duration of Follow-up and Timing of Discharge from Imaging Follow-up, in Adult Patients with Urolithiasis After Surgical or Medical Intervention: A Systematic Review and Meta-analysis from the European Association of Urology Guideline Panel on Urolithiasis. Eur. Urol. Focus..

[43] Lombardo R., Tzelves L., Geraghty R., Davis N.F., Neisius A., Petřík A., Gambaro G., Türk C., Somani B., Thomas K. (2024). Follow-up of urolithiasis patients after treatment: An algorithm from the EAU Urolithiasis Panel. World J. Urol..

[44] Ray A.A., Ghiculete D., Pace K.T., Honey R.J. (2010). Limitations to ultrasound in the detection and measurement of urinary tract calculi. Urology.

[45] Smith-Bindman R., Aubin C., Bailitz J., Bengiamin R.N., Camargo C.A., Corbo J., Dean A.J., Goldstein R.B., Griffey R.T., Jay G.D. (2014). Ultrasonography versus computed tomography for suspected nephrolithiasis. N. Engl. J. Med..

[46] Khan S.R., Canales B.K., Dominguez-Gutierrez P.R. (2021). Randall’s plaque and calcium oxalate stone formation: Role for immunity and inflammation. Nat. Rev. Nephrol..

[47] Vaughan L.E., Enders F.T., Lieske J.C., Pais V.M., Rivera M.E., Mehta R.A., Vrtiska T.J., Rule A.D. (2019). Predictors of Symptomatic Kidney Stone Recurrence After the First and Subsequent Episodes. Mayo Clin. Proc..

[48] Forbes C.M., McCoy A.B., Hsi R.S. (2021). Clinician Versus Nomogram Predicted Estimates of Kidney Stone Recurrence Risk. J. Endourol..

[49] Flammia S., Salciccia S., Tufano A., Busetto G.M., Ricciuti G.P., Sciarra A. (2020). How urinary stone emergencies changed in the time of COVID-19?. Urolithiasis.

